# Semi-Automatic Prostate Segmentation From Ultrasound Images Using Machine Learning and Principal Curve Based on Interpretable Mathematical Model Expression

**DOI:** 10.3389/fonc.2022.878104

**Published:** 2022-06-07

**Authors:** Tao Peng, Caiyin Tang, Yiyun Wu, Jing Cai

**Affiliations:** ^1^Department of Health Technology and Informatics, The Hong Kong Polytechnic University, Hong Kong, Hong Kong SAR, China; ^2^Department of Radiation Oncology, UT Southwestern Medical Center, Dallas, TX, United States; ^3^Department of Medical Imaging, Taizhou People’s Hospital, Taizhou, China; ^4^Department of Medical Technology, Jiangsu Province Hospital, Nanjing, China

**Keywords:** accurate prostate segmentation, transrectal ultrasound, principal curve, constraint closed polygonal segment model, improved differential evolution-based method, machine learning, interpretable mathematical model expression

## Abstract

Accurate prostate segmentation in transrectal ultrasound (TRUS) is a challenging problem due to the low contrast of TRUS images and the presence of imaging artifacts such as speckle and shadow regions. To address this issue, we propose a semi-automatic model termed Hybrid Segmentation Model (H-SegMod) for prostate Region of Interest (ROI) segmentation in TRUS images. H-SegMod contains two cascaded stages. The first stage is to obtain the vertices sequences based on an improved principal curve-based model, where a few radiologist-selected seed points are used as prior. The second stage is to find a map function for describing the smooth prostate contour based on an improved machine learning model. Experimental results show that our proposed model achieved superior segmentation results compared with several other state-of-the-art models, achieving an average Dice Similarity Coefficient (DSC), Jaccard Similarity Coefficient (Ω), and Accuracy (ACC) of 96.5%, 95.2%, and 96.3%, respectively.

## 1 Introduction

Prostate cancer is one of the leading causes of cancer-related deaths among men worldwide (1). Because of the real-time nature and cost-effectiveness, transrectal ultrasound images (TRUS) have become one of the important imaging modalities for physicians to determine the boundaries and volumes of prostates in many treatment procedures, such as prostate brachytherapy (2). However, manual delineation of the prostate boundary is tedious, time-consuming, and often dependent on the training and experiences of radiologists (3). Hence, automatic or semi-automatic prostate segmentation in ultrasound is highly desired to obtain consistent prostate boundaries efficiently for a number of image-guided diagnostic and treatment procedures (4, 5).

**Figure 1:**
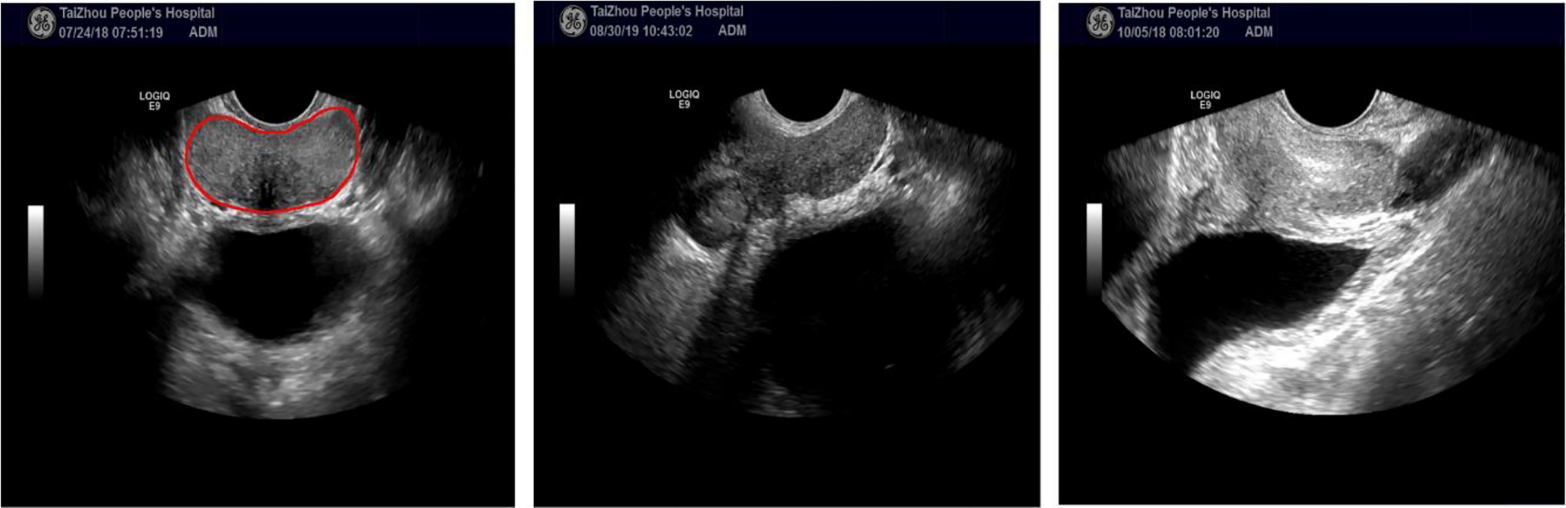
The left image is an example of a TRUS image with a clear prostate boundary. The other two TRUS images show examples with weak or incomplete edge information. All TRUS images are from TaiZhou People’s hospital (see Section 3.1).

Accurate prostate segmentation in ultrasound remains very challenging due to the low contrast of TRUS images, too high image gain, and the presence of imaging artifacts such as speckle, micro-calcifications, and shadow artifacts. Heterogeneous intensity distribution inside the prostate and neighboring tissues further make the prostate segmentation challenging. **Figure 1** illustrates several challenging cases in TRUS prostate automatic segmentation.

In recent years, many different segmentation models have been investigated for medical image segmentation, including: (1) feature classification models (6), (2) region segmentation models (7), and (3) contour detection models (8, 9). Shahedi et al. (10) have proposed a semi-automatic prostate segmentation model in computed tomography (CT) images using local texture classification and statistical shape modeling. The segmentation accuracy of the model is as high as 92%, but the results of the texture extraction part strongly depend on the image resampling accuracy. Ghavami et al. (11) have compared six different convolutional neural networks in segmenting the prostate and discussed the impact of network architecture on the accuracy of volume measurement and MRI-ultrasound registration. On the basis of the deep weakly supervised convolutional neural network (CNN) segmentation proposed in Ref. (12), Kervadec et al. (13) have presented a penalty-based CNN for weakly supervised prostate segmentation and reached a level of segmentation performance that is comparable to full supervision. However, the highest Dice Similarity Coefficient (DSC) value between the full supervision segmentation result and the manually delineated contour is only 0.89. Compared with these data-driven and learning-based models, the shape of the anatomical structure can be obtained by the contour detection model with reduced time and complexity to facilitate prostate segmentation in TRUS.

The key purpose of contour extraction models is to use a shape representation (14) or curve approximation model (15) to denote the organ contour. Balsiger et al. (16) proposed to improve traditional CNN-based volumetric image segmentation through the point-wise classification of point clouds, where the threshold to balance false positives and false negatives is manually selected. Li et al. (17) proposed an active contour model based on adaptive energy weight functions for medical image segmentation with high accuracy. However, the results of the proposed model are affected by the noise and boundary leakage.

Among recent contour extraction models, the principal curve model has gained great interest for detecting abnormal organs from other surrounding normal tissues, due to its ability to effectively deal with noisy input and achieve accurate results (18). In Ref. (19), Alickovic et al. proposed a medical decision support system using principal curve model and random forests classifier for the diagnosis of heart arrhythmia with high accuracy. Meanwhile, Bisele et al. (20) have combined the machine learning model with the principal curve model for assessing human locomotion.

In this work, we propose a novel semi-automatic segmentation framework termed H-SegMod for prostate segmentation on TRUS images. The proposed framework consists of two cascaded stages. In the first stage, using a few radiologist-selected seed points as prior, an improved principal curve model is used to obtain the vertices sequences, which consists of the coordinates of vertices and sequence number of vertices. In the second stage, a smooth prostate contour is obtained by using an Improved Differential Evolution Machine Learning model (IDEML), which combines an improved Differential Evolution model (DE) and a machine learning model.

The key contributions of the proposed method are summarized as follows:

1) We combine the ability of the principal curve method to automatically approach the center of the dataset (21) and use a principal curve-based method to obtain the vertices sequence consisting of vertices ordinates and sequence number of vertices. Compared with other state-of-the-art principal curve models, the Constraint Closed Polygonal Segment model (CCPS) is proposed in our model by adding different normalization strategies and constraint conditions.

2) A mathematical map model (realized by the IDEML) is found to describe the smooth prostate contour represented by the output of the neural network (i.e., optimized vertices) that can match the ground truth contour. By using IDEML for training, the model error can be minimized to obtain an accurate segmentation result.

3) The IDEML consists of an Improved Adaptive Mutation and Crossover-based Differential Evolution model (IAMCDE) with an Adaptive Learning-Rate Back Propagation Neural Network (ABPNN), where the IAMCDE is proposed using different mutation steps and loop constraint conditions as compared with other state-of-the-art differential evolution models.

The remainder of this paper is organized as follows. Section 2 presents the proposed model, Section 3 describes the dataset and performance metrics, and Section 4 presents the results. Section 5 draws a discussion and conclusion

## 2 Model

### 2.1 Overview of the Proposed Model

**Figure 2:**
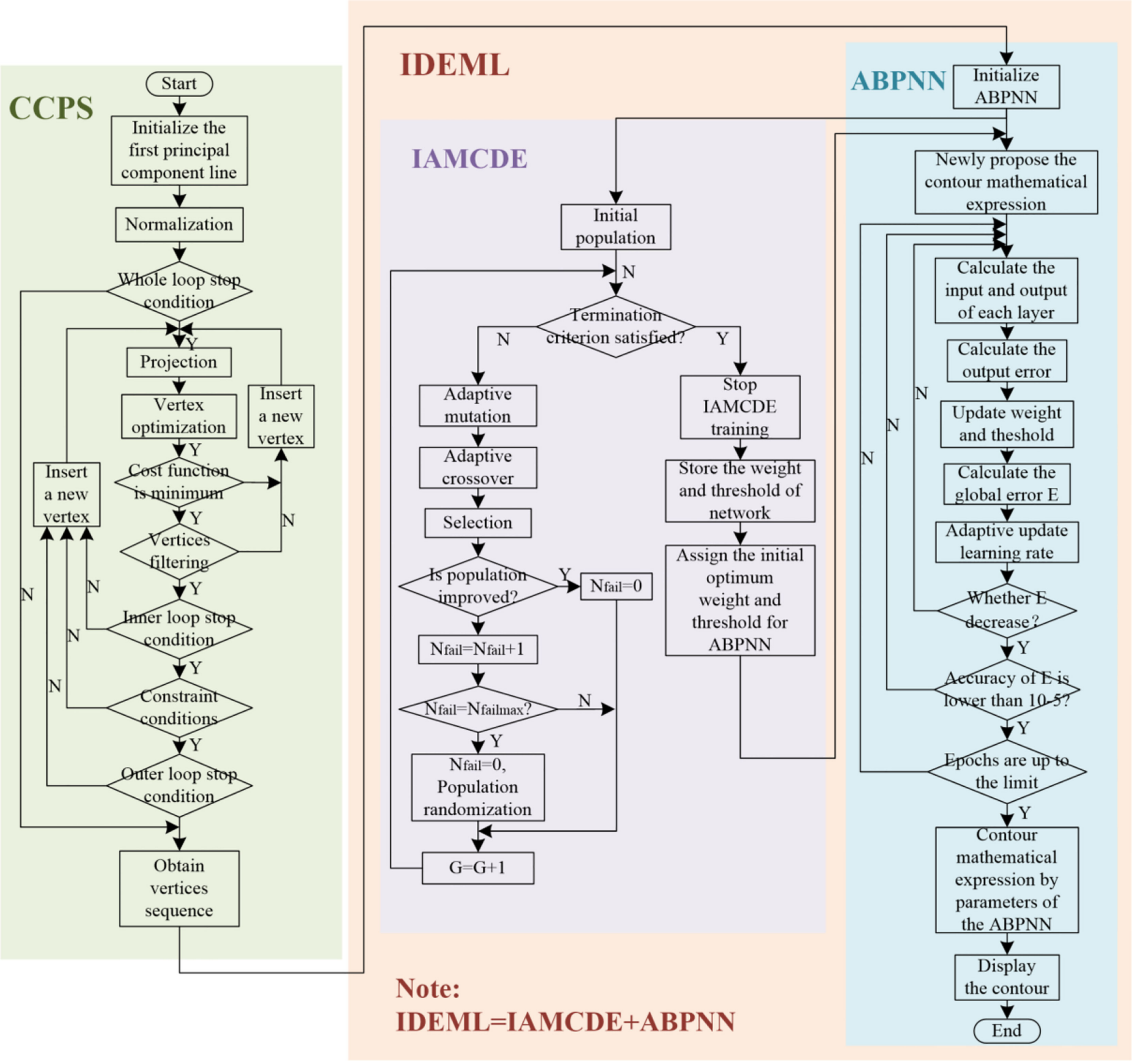
The flowchart of the proposed model, including the CCPS and IDEML, where the IDEML consists of the IAMCDE and the ABPNN.

The proposed H-SegMod consists of two main components: 1) improved principal curve model and 2) improved enhanced neural network. The flowchart of our H-SegMod is shown in **Figure 2**. The goal of this proposed model is to reduce the workload of expert radiologists and obtain smooth and accurate prostate contours. We first use a few radiologist-selected seed points as prior. Then CCPS is used for obtaining the vertices sequences, which consists of the sequence number of vertices, and their corresponding coordinates. Next, we used the IDEML for training, where the sequence number of vertices is used as the input of IDEML, and vertices’ coordinates are used to minimize the mean square error. Finally, after training, the parameters of the IDEML are used to denote the map function for expressing the smooth mathematical model of prostate contour. Furthermore, we add the input/output of each step of the proposed method in **Table 1**.

**Table 1:** Input/output of each step of the proposed method.

Method	Input	Output
CCPS	Seed points	vertices sequences (sequence number of vertices and their corresponding coordinates)
IAMCDE	Initial parameters (shown in Section 2.3.1)	Optimal initial weight and threshold of ABPNN
ABPNN	Vertices sequences	Prostate contour

### 2.2 Constraint Closed Closed Polygonal Segment Model (CCPS)

Principal curve was first proposed by Hastie & Stuetzle (21) as a smooth one-dimensional curve that passes through the middle of an *n*-dimensional data set, providing a nonlinear summary of the data. To improve the efficiency and robustness of our previous work named Closed Polygonal Segment model (CPS), we proposed the CCPS.

#### 2.2.1 CPS

According to the concept of principal curve (21), Kegl et al. designed the K-segments Principal Curve method (KPC) (22) to find the *k*-segment principal curve. Considering that the traditional KPC cannot handle the issue of the unfitted closed dataset, we have previously proposed the CPS (23–25). To make the improvement of CPS intuitive, compared with KPC, we list the added constraints and stop conditions.

##### 2.2.1.1 Constraints

Our improvements are from curve shape-based, segments-based, and vertices-based parts.

During the procedure of finding the principal curve, the curve of the prostate contour keeps closed. In addition, we will retain the longest segment so that it can include the greatest number of projection points.

To find an optimal vertex, the main steps of selection of the optimal vertex are shown as follows: firstly, inserting a new vertex, the data point set is projected in the segments or the determined vertices. Secondly, the Euclidean distance from points to the curve of the contour is obtained. Finally, the position of each vertex is updated only if the value of the distance function is smaller.

##### 2.2.1.2 Stop Conditions

Our previously proposed stop conditions contain the whole loop-based, outer loop-based, and inner loop-based parts.

If the number of segment *is* satisfies the condition, the whole loop will stop, where the whole loop condition is shown in Eq. (1).

(1) $$\text{is}>\text{β}{\text{n}}^{1/3}{\text{Δ}}_{\text{n}}{\left({\text{f}}_{\text{k},\text{n}}\right)}^{-1/2}\text{r}$$

The parameter β equals 0.3, which is determined by several trial runs (22), n shows the number of data points, f denotes the principal curve, and r is the radius of data points.

Both the inner and outer loops should meet the condition that the difference in value between the current distance and the last loop distance is smaller than the maximum distance deviation Δs=0.002.

#### 2.2.2 CCPS

To deal with the issue that the practical usefulness of both KPC and CPS is severely affected by issues of sparse, uneven distributions, and abnormal data. Furthermore, the performance of the KPC suffers from the dependencies of predetermined parameters for the underlying optimization. In this work, we propose CCPS to deal with these issues, which combines several modified constraints of the Constraint K-segment Principal Curve model (CKPC) that were originally proposed in Ref (26). The refinements of CCPS mainly include two parts: 1) different initialization or normalization strategies and 2) vertices filtering.

##### 2.2.2.1 Improved Initialization

In Ref. (26), Zhang et al. introduced two points p_1_ and p_2_ from the neighborhood of the “true” curve to construct a first principal component line, where p_1_ and p_2_ are the start point and endpoint of the first principal component line, respectively. In this work, considering that the KPC (22) and CKPC (26) cannot deal with the closed dataset well, we use a different initialization strategy. A closed square containing all the projection points is used as the first principal component line, where the coordinates of the vertices of the closed square are (0.1, 0.1), (-0.1, 0.1), (-0.1, -0.1), (0.1, -0.1), and (0.1, 0.1), respectively.

##### 2.2.2.2 Improved Normalization

As our previous works use the Min-Max normalization method (27) with the weak anti-interference ability, we adopt an improved normalization strategy by using the Z-score standardization method (28). The mean μ and variance σ are defined in Eq. (2) and Eq. (3), respectively.

(2) $${\text{μ}}_{\text{x}}=\frac{1}{\text{n}}\sum_{\text{i}=1}^{\text{n}}{\text{x}}_{\text{i}}\text{ and }{\text{μ}}_{\text{y}}=\frac{1}{\text{n}}\sum_{\text{i}=1}^{\text{n}}{\text{y}}_{\text{i}}$$

(3) $${\text{σ}}_{\text{x}}=\sqrt{\frac{1}{\text{n}}\sum_{\text{i}=1}^{\text{n}}\left[{\left({\text{x}}_{\text{i}}-{\text{μ}}_{\text{x}}\right)}^{2}\right]}\text{ and }{\text{σ}}_{\text{y}}=\sqrt{\frac{1}{\text{n}}\sum_{\text{i}=1}^{\text{n}}\left[{\left({\text{y}}_{\text{i}}-{\text{μ}}_{\text{y}}\right)}^{2}\right]}$$

where x_i_ and y_i_ are the x-axis coordinate and y-axis coordinate of point p_i_, respectively.

The new x-axis coordinate x_i_^'^ and y-axis coordinate y_i_^'^ are calculated in Eq. (4).


(4) $${\text{x}}_{\text{i}}^{\prime }=\frac{{\text{x}}_{\text{i}}-{\text{μ}}_{\text{x}}}{{\text{σ}}_{\text{x}}}\;\text{and}\;{\text{y}}_{\text{i}}^{\prime }=\frac{{\text{y}}_{\text{i}}-{\text{μ}}_{\text{y}}}{{\text{σ}}_{\text{x}}}$$

##### 2.2.2.3 Vertices Filtering

In the vertex optimization step, the position of each vertex is based on the principle that the distance from the sample point to the principal curve is the smallest (29). If the optimization fails, the locations of some vertices may produce a distorted principal curve. To deal with this issue, we introduced a constraint term to verify and remove any abnormal vertices after the vertex optimization step. The flag of removing vertices is defined in Eq. (5).

(5) $$\text{Flag}\left({\text{v}}_{\text{i}}\right)=\{\begin{array}{l} 1,\\ 0, \end{array}\begin{array}{c} \text{if}\left\{\left(1_{{\text{s}}_{i-1}}\text{or }1_{\text{s}}\right)>\text{r}\right\}\\ \text{otherwise} \end{array}$$

where 1_s_i__ is the length of the i-th line segment and satisfies 1_s_i__=∥v_i+1_−v_i_ ∥1≤i≤n, r is the radius of data and satisfies $\text{r}=\text{max}\text{x}\in \text{V}∥\text{x}-\frac{1}{\text{n}}\sum \text{y}\in \text{V}\text{y}∥$ , n is the total number of vertices in dataset V, x and y are the x-axis and y-axis coordinate of the vertex v_i_, respectively.

If Flag(v_i_)=1, we keep the vertex, and if Flag(v_i_)=0, we delete the vertex. There are two situations in which the vertex v_i_ must be removed:1) The vertex goes outside the radius of the data, and 2) the number of data points projected onto the vertices and neighboring segments is less than five in this work.

### 2.3 Improved Differential Evolution Machine Learning Model (IDEML)

When a Neural Network (NN) randomly initializes the network connection weights and thresholds, the training results can be potentially trapped into the local minimum instead of the global optimum. As such, the final training results of the learning algorithm strongly depend on the initial weights and thresholds (30). Due to the good ability of the Differential Evolution model (DE) for global search, many studies use DE or an improved version of DE for preliminarily searching for the initial optimal connection weights and thresholds of NN (31, 32). In this work, we propose a new IDEML that combines the IAMCDE with ABPNN. The vertices sequences obtained by the CCPS are used by the IDEML. Before the ABPNN is ready to train, we use the IAMCDE to search the initial optimal connection weights and thresholds of ABPNN. After the ABPNN has completed training, we can obtain the ABPNN’s optimal parameters, such as weights and thresholds. The parameters of the ABPNN are used to express the mathematical expression of prostate contour, which is shown in Section 2.4.

#### 2.3.1 Improved Adaptive Mutation and Crossover-Based Differential Evolution Model (IAMCDE)

The traditional Differential Evolution model (DE) proposed by Storn et al. (33) has been used as a global search technique. However, both mutation Factor (F) and Crossover Rate (CR) with a fixed value will affect the convergence speed and the quality of the solution. Zeng et al. (30) have proposed an Adaptive Mutation and Crossover-based Differential Evolution model (AMCDE) and validated it on the energy consumption prediction. Considering that the AMCDE can be trapped into the local optimum, our work proposes an improved AMCDE (IAMCDE). The refinements of IAMCDE mainly include two parts: 1) two mutation operators and 2) a new global optimum search approach based on a population randomization strategy.

##### 2.3.1.1 Traditional DE

The details of the key steps are briefly described as follows:

(1) Initialization: The main parameters, such as population size S, mutation factor F, crossover rate CR, the range of search space [U_min_, U_max_], and length of chromosome CS, are initialized. The initial population pop_ij_ is randomly created in Eq. (6).

(6) $${\text{pop}}_{\text{sj}}={\text{U}}_{\text{min}}+\text{rand }[0,1]\;\times \;\left({\text{U}}_{\text{max}}-{\text{U}}_{\text{min}}\right)$$

where s=1,2,.,S and rand (0, 1) is to generate a random number between 0 and 1.

(2) Mutation: For each candidate ${\text{pop}}_{\text{s}}^{\text{G}},\;s = 1, 2,\ldots \text{ S}$ , the DE generates a new corresponding mutated individual, which is denoted in Eq. (7).

(7) $${\text{npop}}_{\text{s}}^{\text{G}+1}=\text{po}{\text{p}}_{\text{p}{\text{m}}_{1}}^{\text{G}}+\text{F}\times \left(\text{po}{\text{p}}_{\text{p}{\text{m}}_{2}}^{\text{G}}-\text{po}{\text{p}}_{\text{p}{\text{m}}_{3}}^{\text{G}}\right)$$

where the population members pm_1_, pm_2_, and pm_3_ are randomly selected, with each of them being different. F is used to control the mutation process, which is distributed in the range of [0, 2], as discussed in Ref (33, 34).

(3) Crossover: The crossover step mixes the mutated vectors and the target vectors to increase the variety of the parameter vector. The rule is shown in Eq. (8).

(8) $${\text{u}}_{\text{sj}}^{\text{G}+1}=\{\begin{array}{r} \text{npo}{\text{p}}_{\text{sj}}^{\text{G}+1},\\ \text{po}{\text{p}}_{\text{sj}}^{\text{G}}, \end{array}\;\begin{array}{c} \text{if pm(j)}\leq \text{CR}\\ \text{otherwise} \end{array}$$

(4) Selection: The trial individual (offspring) produced by the crossover operator is compared with the target individual (parent). Whether the individual will be a part of the next generation is determined by the following selection method [Eq. (9)].


(9) $${\text{pop}}_{\text{s}}^{\text{G}+1}=\{\begin{array}{cc} {\text{u}}_{\text{s}}^{\text{G}+1}, & \text{if f}\left({\text{u}}_{\text{s}}^{\text{G}+1}\right)<\text{f}\left({\text{pop}}_{\text{s}}^{\text{G}}\right)\\ {\text{pop}}_{\text{s}}^{\text{G}}, & \text{otherwise} \end{array}\;$$

##### 2.3.1.2 Previously proposed AMCDE

To improve the convergence speed and the quality of the solution, the main improvements of Ref (30). are shown in Eq. (10) and Eq. (11).

(10) $$\text{F}=\{\begin{array}{l} \text{a}+\left(1-\text{a}\right)\times \text{sin}\left(\frac{\text{G}}{\text{GMax}}\text{π}-\frac{1}{2}\text{π}\right),\text{if G}\leq \frac{\text{GMax}}{2}\\ \text{a}-\left(1-\text{a}\right)\times \text{cos}\left(\frac{1}{2}\text{π}-\frac{\text{G}}{\text{GMax}}\text{π}\right),\text{ otherwise} \end{array}$$

(11) $$\text{CR}=\{\begin{array}{l} \text{b}+\left(1-\text{b}\right)\times \text{sin}\left(\frac{\text{G}}{\text{GMax}}\text{π}-\frac{1}{2}\text{π}\right),\text{if G}\leq \frac{\text{GMax}}{2}\\ \text{b}-\left(1-\text{b}\right)\times \text{cos}\left(\frac{1}{2}\text{π}-\frac{\text{G}}{\text{GMax}}\text{π}\right),\text{ otherwise} \end{array}$$

where a and b are constants within the range [0.5, 1], GMax is the maximum iteration number, and G is the present iteration number.

At the beginning and end stages, both F and CR change slower, where sine function and cosine function are in a 1/4 cycle with a value within [1, 0], respectively. In the middle stage, they change relatively faster. The improvements of AMCDE have shown good performance in finding the optimal global solution (30).

##### 2.3.1.3 Our Newly Proposed IAMCDE

In this work, we propose a new IAMCDE by adding two new improvements based on the AMCDE (30).

First, we use two mutation operators to generate mutant vectors according to Eq. (12).

(12) $${\text{npop}}_{\text{i}}^{\text{G}}=\{\begin{array}{cc} {\text{pop}}_{{\text{i}}_{1}}^{\text{G}}+{\text{rand}}_{1}\times \left({\text{pop}}_{{\text{i}}_{2}}^{\text{G}}-{\text{pop}}_{{\text{i}}_{3}}^{\text{G}}\right), & \text{if and }\left[0,1\right]><{\text{P}}_{\text{G}}\\ {\text{pop}}_{{\text{i}}_{1}}^{\text{G}}+{\text{rand}}_{2}\times \left({\text{pop}}_{{\text{i}}_{2}}^{\text{G}}-{\text{pop}}_{{\text{i}}_{3}}^{\text{G}}\right)+{\text{rand}}_{3}\times \left({\text{pop}}_{{\text{i}}_{4}}^{\text{G}}-{\text{pop}}_{{\text{i}}_{5}}^{\text{G}}\right), & \text{otherwise} \end{array}$$

where i_k_ (k=1,2,3,4,5) represent five random integer numbers in the range of [1, N_p_] and N_p_ is the number of solutions; rand1, rand2, rand3, and rand[0,1] are the four random numbers in the range of [0, 1]. Random numbers are used to produce mutation factors in each generation. During the evolution process, a mutation factor can be selected in the range of [0, 1], which is useful for both global and local search. p_G_ shows the probability of using the mutation operator and is updated in Eq. (13).

(13) $${\text{pro}}_{\text{G}}={\text{pro}}_{\text{min}}+\frac{\text{G}\times \left({\text{pro}}_{\text{max}}-{\text{pro}}_{\text{min}}\right)}{\text{GMax}}$$

where pro_max_ and pro_min_ are the maximal and minimal probability of using the mutation operator.

Second, we propose a new global optimum search approach based on a population randomization strategy to help IAMCDE to avoid the local optimum. The IAMCDE may get trapped in the optimum local search, which causes the search space to be limited. Here, the execution step of the new global optimum search approach is shown as follows: we set the current number of consecutive failures N_fail_=0 and the max number of consecutive failures N_failmax_. If the current population is improved in the Gth generation, we set N_fail_=0. Otherwise, N_fail_=N_fail_+1. When N_fail_=N_failmax_, we will set N_fail_=0 and carry out the population randomization strategy as follows: First, we sort all solutions of the population by decreasing order according to their function values. Second, the first N_P_/2 solutions are reserved for the next generation, and the remaining N_P_/2 solutions are removed from the population. Finally, N_P_/2 new solutions are added to the population, which are generated according to Eq. (6).

#### 2.3.2 Adaptive Learning-Rate Back Propagation Neural Network (ABPNN)

Using NN for training using vertices sequence obtained by the CCPS, the model error can be minimized to refine the segmentation results. However, there are many drawbacks in the training step, i.e., slow convergence, easy to get trapped to local minima. In this work, we propose the ABPNN. The main improvement of ABPNN is using the adaptive learning rate. In traditional BPNN, the learning rate is constant. However, the learning rate directly affects the convergence efficiency of the machine learning model. When the learning rate is lower, it needs more training time, and the convergence of the model becomes slower. When the learning rate is too high, the model will become unstable due to oscillation and divergence. Therefore, the trend of the model error E is updated in Eq. (14), which is according to the following strategy: 1) If the model error E decreases from the previous iteration, the learning rate increases; and 2) If the model error E increases from the previous iteration, the learning rate decreases.

(14) $${\text{η}}_{\text{k}}=\{\begin{array}{cc} \text{α}\times {\text{η}}_{\text{k}-1} & {\text{E}}_{\text{k}}<{\text{E}}_{\text{k}-1}\\ \text{β}\times {\text{η}}_{\text{k}-1} & {\text{E}}_{\text{k}}>{\text{E}}_{\text{k}-1}\\ {\text{η}}_{\text{k}-1} & \text{others} \end{array}$$

where k is the epochs at the training step, E is the error function and $\text{E}=\sum \text{i}=1\text{n}{\left({\text{y}}_{\text{ic}}-{\text{y}}_{\text{id}}\right)}^{2}$ . y_ic_ shows the actual result, and y_id_ is the desired result. α is the constant within the range (1, 2) and β is the constant within the range [0, 1], where we set α and β at 1.5 and 0.5 in this work, respectively

### 2.4 Interpretable Mathematical Expression of Prostate Contour

Due to the feed-forward neural network with one hidden layer used to approximate any continuous function, we choose the ABPNN with only one hidden layer. Meanwhile, we use the three-layer ABPNN with two output neurons in the output layer, where the parameters of the ABPNN are used to express the values of two output neurons. Then, the values of two output neurons are used to denote the x-axis coordinate and y-axis coordinate of the contour points, respectively. Finally, the prostate contour can be obtained by the contour points.

In this work, we use the parameters of the ABPNN to express the values of two output neurons, shown in Eq. (15).

(15) $$({\text{g}}_{\text{x}}(\text{t}),{\text{g}}_{\text{y}}(\text{t}))=(\begin{array}{c} \frac{{\text{e}}^{\sum_{j=1}^{\text{k}}\frac{1}{{1+e}^{\sum_{i=1}^{\text{q}}{\text{-(tω}}_{\text{i}}{\text{-T}}_{\text{i}})}}{\text{a}}_{j,1}{\text{-b}}_{j,1}}{\text{-e}}^{-\sum_{j=1}^{\text{k}}\frac{1}{{1+e}^{\sum_{i=1}^{\text{q}}{\text{-(tω}}_{\text{i}}{\text{-T}}_{\text{i}})}}{\text{a}}_{j,1}{\text{-b}}_{j,1}}}{{\text{e}}^{\sum_{j=1}^{\text{k}}\frac{1}{{1+e}^{\sum_{i=1}^{\text{q}}{\text{-(tω}}_{\text{i}}{\text{-T}}_{\text{i}})}}{\text{a}}_{j,1}{\text{-b}}_{j,1}}{\text{+e}}^{-\sum_{j=1}^{\text{k}}\frac{1}{{1+e}^{\sum_{i=1}^{\text{q}}{\text{-(tω}}_{\text{i}}{\text{-T}}_{\text{i}})}}{\text{a}}_{j,1}{\text{-b}}_{j,1}}},\\ \frac{{\text{e}}^{\sum_{j=1}^{\text{k}}\frac{1}{{1+e}^{\sum_{i=1}^{\text{q}}{\text{-(tω}}_{\text{i}}{\text{-T}}_{\text{i}})}}{\text{a}}_{j,2}{\text{-b}}_{j,2}}{\text{-e}}^{-\sum_{j=1}^{\text{k}}\frac{1}{{1+e}^{\sum_{i=1}^{\text{q}}{\text{-(tω}}_{\text{i}}{\text{-T}}_{\text{i}})}}{\text{a}}_{j,2}{\text{-b}}_{j,2}}}{{\text{e}}^{\sum_{j=1}^{\text{k}}\frac{1}{{1+e}^{\sum_{i=1}^{\text{q}}{\text{-(tω}}_{\text{i}}{\text{-T}}_{\text{i}})}}{\text{a}}_{j,2}{\text{-b}}_{j,2}}{\text{+e}}^{-\sum_{j=1}^{\text{k}}\frac{1}{{1+e}^{\sum_{i=1}^{\text{q}}{\text{-(tω}}_{\text{i}}{\text{-T}}_{\text{i}})}}{\text{a}}_{j,2}{\text{-b}}_{j,2}}}) \end{array}$$

The corresponding parameters are denoted as follows:

g(•) denotes the value of the output unit; t denotes the vertices sequence; q is the number of hidden neurons; w_i_(i=1,2,…,q) is the weight from the input layer to the i-th hidden neuron; T_i_(i=1,2,…,q) is the output threshold of the i-th hidden neuron; a_i,k_(i=1,2,…,q; k=1,2) is weight from the i-th hidden neuron to the k-th output neuron; b_k_(k=1,2) is the output threshold of the k-th neuron at the output layer;

The proposed mathematical expression of prostate contour can be obtained in Eq. (16).

(16) $$\text{f}(\text{t})=\left(\text{x}(\text{t)},\text{y}(\text{t})\right)=\left(\frac{{\text{g}}_{\text{x}}\text{(t)}+1-\sqrt{1-({\text{g}}_{\text{x}}{\text{(t})}^{2}}}{2*{\text{g}}_{\text{x}}\text{(t)}},\frac{{\text{g}}_{\text{y}}\text{(t)}+1-\sqrt{1-{\left({\text{g}}_{\text{y}}\text{(t})\right)}^{2}}}{2*{\text{g}}_{\text{y}}\text{(t)}}\right)$$

where x(t) denotes the x-axis coordinate of the contour points, and y(t) denotes the y-axis coordinate of the contour points.

## 3 Experimental Setup

### 3.1 Dataset

A dataset consisting of 100 brachytherapy patients is used to evaluate the proposed model. All images were obtained at the Taizhou People’s Hospital, Jiangsu, China. The study protocol was reviewed and approved by the Ethics Committee of our institutional review board, and informed consent was obtained from all patients.

All TRUS data were obtained using the General Electric LOGIQ E9 (LE9) system and an integrated TRUS probe with a frequency in the range of 5-8 MHz. The age of the patients ranged from 18 to 56. All images are in DICOM file format, which has a matrix size of 700×615 pixels.

The ground truths prostate contours are marked and verified by five expert radiologists. All the expert radiologists have over 15 years experience in dealing with prostate anatomy, prostate segmentation, and ultrasound-guided biopsies. Each expert radiologist independently checks their own marks along with the anonymous marks of the other radiologists, and the consensus ground truths are obtained by the majority voting of five experts’ annotations.

### 3.2 Performance Metrics

To demonstrate the performance of the proposed model, we use Dice Similarity Coefficient (DSC) (2), Jaccard Similarity Coefficient (Ω) (3), and Accuracy (ACC) (6) as the evaluation metrics. The metrics are shown in Eq. (17), Eq. (18), and Eq. (19), respectively.

(17) $$\text{Ω}=\frac{\text{TP}}{\text{FP+TP+FN}}$$

(18) $$\text{DSC=}\frac{2TP}{2TP+FP+FN}$$

(19) $$\text{ACC=}\frac{\text{TP+TN}}{\text{TP+FN+FP+TN}}$$

where TP, FP, FN, and TN represent True Positive, False Positive, False Negative, and True Negative, respectively.

## 4 Results

In this section, we describe the series of experiments used to evaluate the proposed model. The dataset (100 patients, 605 images) was randomly divided into three groups, where one group with 50 patients (350 images) was used for training, one group with 25 patients (100 images) was used for validation, and the other group with 25 patients (155 images) was used for testing. First, the training and validation sets were used to select the optimal parameters of the proposed model (Section 4.1). Second, the accuracy and stability of the proposed model were further evaluated using different evaluation metrics on the testing set. Considering that different patients may have different numbers of slices, the average values over all slices from the same patient are calculated for each patient (Section 4.2). Third, we compared the proposed model with several hybrid models quantitatively and qualitatively (Section 4.3). Then, the curve fitting model was selected to compare with our proposed model (Section 4.4). Finally, we compared our proposed model with several other state-of-the-art models (Section 4.5). All experiments have been performed on a computer with Intel Core i7-8750H CPU and Geforce GTX 1070 GPU with 8G memory.

### 4.1 Selecting the Best Performance of the Proposed Model

#### 4.1.1 Comparison on Different Neurons

**Figure 3:**
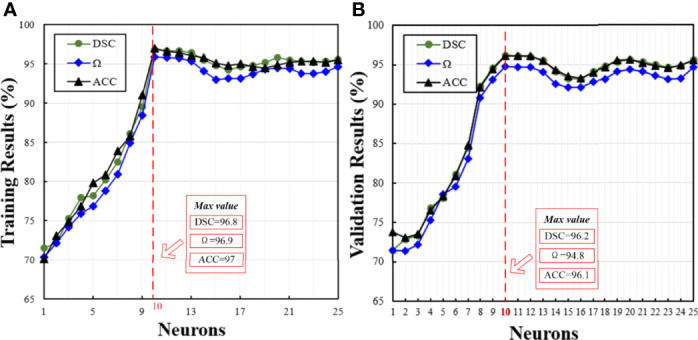
Corresponding training and validation results at different hidden neurons. Green, blue, and black curves show the changes of the DSC, Ω, and ACC, with the number of neurons, respectively. Considering that all the metrics have nearly the same trend and obtain the max value at the same neurons, we use the red dotted line to represent the position of obtaining the max values of all the metrics. In this figure, **(A, B)** show the training and validation results under different neurons, respectively.

**Figure 3** shows the training and validation results obtained by the proposed model on different hidden neurons. The number of epochs is set to 1000. Overall, the influence of the number of neurons on training results is similar to that of the validation results. The performance measured by three metrics, i.e., DSC, Ω, and ACC, shows the same trend as the number of neurons increases. When the neuron number is 1, all the training results are as low as 70%, and the validation results are slightly higher than the training results. The possible reason is that the complex problem fails to be handled with the ABPNN, when the neurons are not enough at the hidden layer. When the neuron number is 10, the model achieves the best performance, where the DSC, Ω, and ACC of validation results are 96.2%, 94.8%, and 96.1%, respectively. As the number of neurons further increases, the model performance starts to degrade. These results suggest that too many hidden neurons may cause overfitting with increased training time.

#### 4.1.2 Comparison of Different Epochs

**Figure 4:**
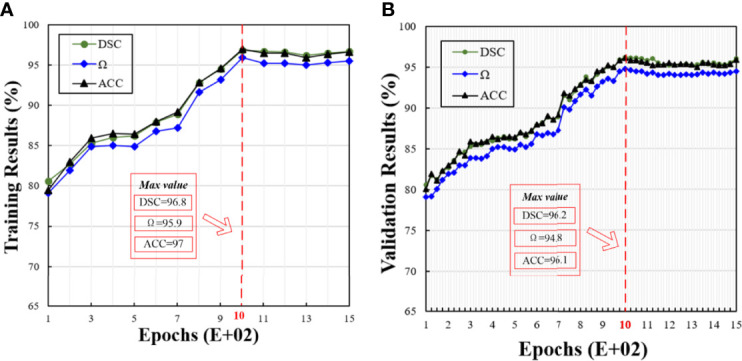
Corresponding training and validation results at different epochs. In this figure, **(A, B)** show the training and validation results under different epochs, respectively.

Based on the results obtained in the previous session, we used 10 neurons in the remaining experiments. **Figure 4** shows the training and validation results in different epochs, where the trend between the training and validation results is similar. With the increasing epochs, all the metrics, i.e., DSC, Ω, and ACC, are increasing. When the epoch is 1000, the model achieves the best performance. After 1000 epochs, the model performance becomes stable. Therefore, we set it at 1000 epochs.

### 4.2 Comparison With Different Metrics on Different Patients

**Figure 5:**
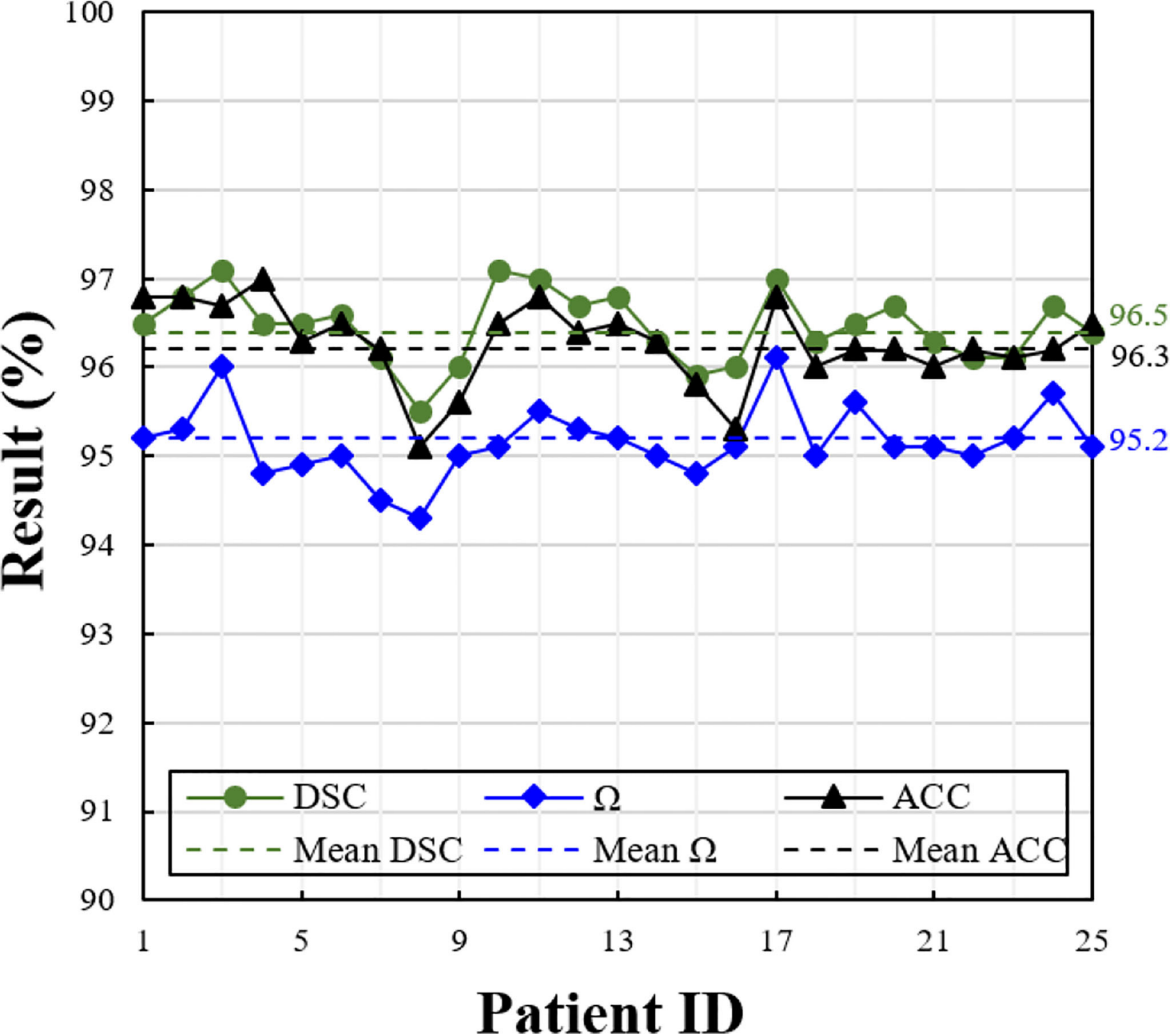
Performance measures of the proposed model at different evaluation metrics (i.e., DSC, Ω, and ACC), on the testing set consisting of 25 patients. The solid line shows the value of each patient, and the dotted line shows the average value of whole patients.

Based on the selected optimal model in the previous section, we evaluated the model performance on the testing set. The metrics of each patient of the testing set are shown in **Figure 5**. Due to different patients having a different number of slices, we show the average value of the metrics on each patient. Overall, the proposed model achieves good segmentation performance evaluated by three different metrics. The average DSC, Ω, and ACC are 96.5%, 96.3%, and 95.2%, respectively.

### 4.3 Comparison With Hybrid Models

In this section, the proposed H-SegMod segmentation model is compared with three other hybrid models qualitatively and quantitatively. Three different metrics, i.e., DSC, Ω, and ACC, are used for evaluation, and the description of the three compared models is summarized below:

Hybrid model 1 is the Closed Polygonal Segment model with -Differential Evolution-Back Propagation Neural Network (CPS-DE-BPNN). Hybrid model 1 combines the strategies presented in Ref. (25) and Ref. (33) to complete the prostate segmentation.

Hybrid model 2 is the CPS-Adaptive Mutation and Crossover-based Differential Evolution model coupled with -BPNN (CPS-AMCDE-BPNN). On the basis of Hybrid model 1, Hybrid model 2 was improved by combining with the strategy proposed in Ref (30).

Hybrid model 3 is the Constraint Closed Polygonal Segment model with -AMCDE and -Back Propagation Neural Network (CCPS-AMCDE-BPNN). Compared with the Hybrid model 2, Hybrid model 3 uses the improved CCPS.

All the models are semi-automatic and use the same training, validation, and testing sets. We set 1000 epochs and 10 neurons for all the models. Based on our previous studies (25), we set the learning rate of the three compared models with a constant at 0.4. Due to the self-adaptive learning rate of our proposed model, our proposed model only needs to set the initial learning rate at 0.5.

**Figure 6:**
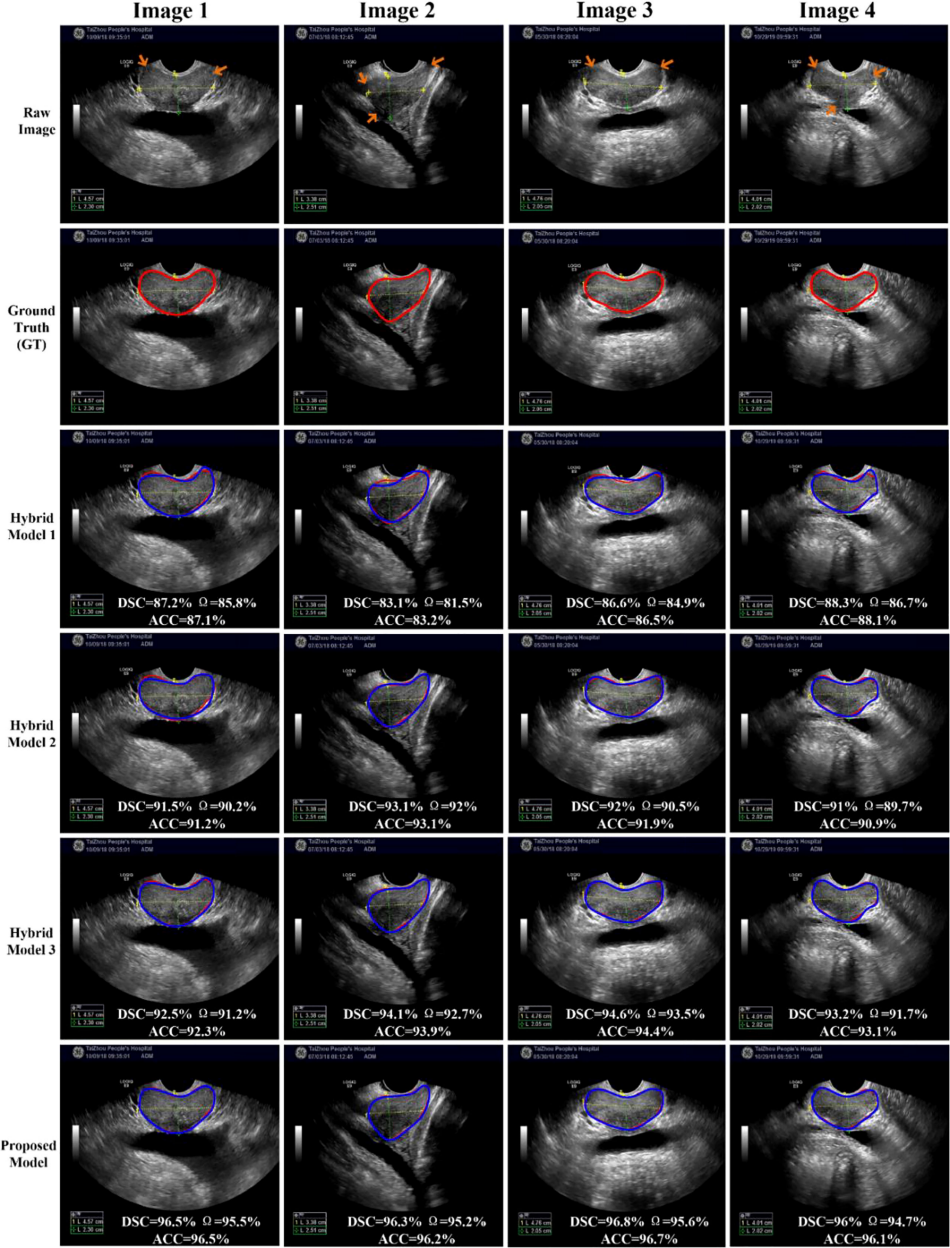
Visual comparison of prostate segmentation results.

To compare the performance of all the hybrid models, we test all the hybrid models on the whole testing set quantitatively. The results of these Hybrid models are shown in **Table 2**, whereas the results of our proposed H-SegMod are shown in the previous section. Meanwhile, we randomly selected four TRUS results (Image 1-Image 4) for visual comparison, as shown in **Figure 6**. The first row in **Figure 6** denotes raw prostate TRUS images. As the prostate TRUS images often contain the missing/ambiguous boundaries (shown in orange arrows), the expert radiologists first mark the approximate range of the prostate with yellow and green labels (shown in raw images). The second row denotes Ground Truth (GT) delineated by expert radiologists. The last four rows show the experimental results of the four models, where red lines show the GT and blue lines show the segmentation result.

**Table 2:** Quantitative comparison with hybrid models.

Model	Method	Model	DSC (%)	Ω (%)	ACC (%)
Hybrid model 1	CPS-DE-BPNN	Hybrid	91.6	90.6	91.8
Hybrid model 2	CPS-AMCDE-BPNN	Hybrid	92.1	90.3	92.1
Hybrid model 3	CCPS-AMCDE-BPNN	Hybrid	93.8	92.6	93.7
Proposed model	H-SegMod	Hybrid	96.5	95.2	96.3

Overall, the proposed model has the best performance.

### 4.4 Comparison With Curve Fitting Model

**Figure 7:**
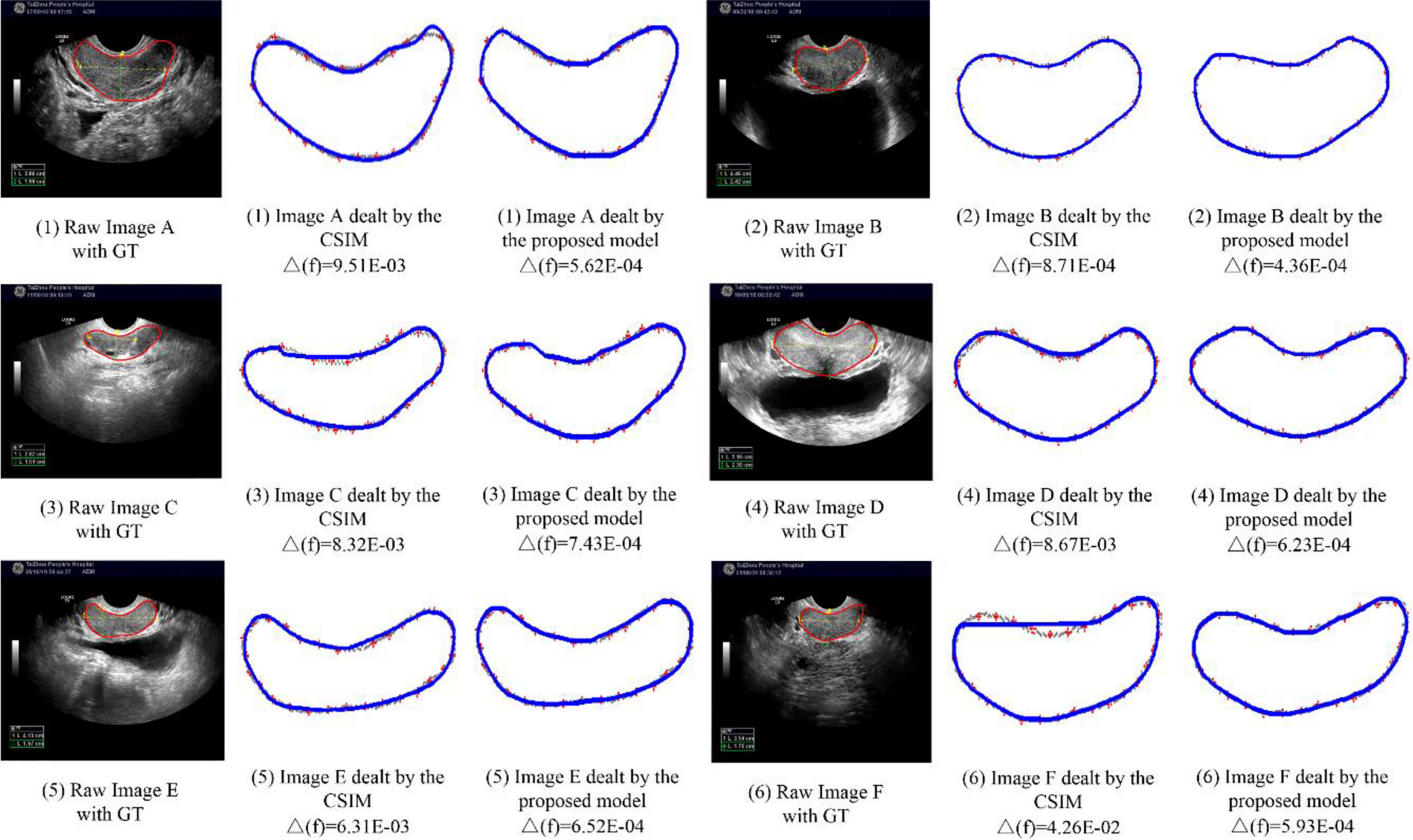
Comparison between CSIM and proposed model.

In this section, we compare our proposed H-SegMod with the cubic spline interpolation model (CSIM) quantitatively and qualitatively, where the CSIM is a curve fitting model. The CSIM is firstly proposed by Mckinley et al. (35) and extended to the medical field (36, 37). We test both models on the whole testing set, and the results are summarized in **Table 3**. Both models only use a few radiologists-defined seed points as initial points. Meanwhile, six images are randomly selected from the testing set, which is different from the four images qualitatively evaluated in the previous section. The visual comparison results between CSIM and our proposed model are shown in **Figure 7**.

**Table 3:** Quantitative comparison with CSIM.

**Method**	**Model**	**DSC (%)**	**Ω (%)**	**ACC (%)**
CSIM	Curve fitting	94.3	93.1	94.2
H-SegMod	Hybrid	96.5	95.2	96.3

In **Figure 7**, the contours obtained by the proposed model are closer to the GT contours than CISM in most of the cases. We further quantify the performance difference by the Euclidean square distance function denoted by Δf. With a smaller Δf, the principal curve f(t) is closer to the initial dataset. Compared with CSIM, the Δf of the proposed model is smaller, which means the proposed model achieves higher accuracy of contoured prostate.

### 4.5 Comparison With Other State-of-the-Art Models

We further evaluated the proposed H-SegMod on the whole testing set against two widely used hybrid approaches, including CPL-BNNM (25) and Hull-CPLM (24), and two deep learning-based approaches, including Unet (38) and Mask-RCNN (39). Two deep learning networks are the automatic segmentation models, and the three hybrid models, including our proposed model, are the semi-automatic models. The results of these experiments are presented in **Table 4**.

**Table 4:** Quantitative comparison with other state-of-the-art models.

Paper	Method	Model	DSC (%)	Ω (%)	ACC (%)
(38)-2015	Unet	Deep learning	91.3	89.6	90.7
(39)-2017	Mask-RCNN	Deep learning	93.1	92.4	92.9
(25)-2018	CPL-BNNM	Hybrid	91.8	90.5	91.4
(24)-2019	Hull-CPLM	Hybrid	94.9	94.3	94.6
Proposed model	H-SegMod	Hybrid	96.5	95.2	96.3

Due to the accuracy of deep learning-based models being affected by the number of limited training data (40), we augmented raw training data (350 slices), where each slice was rotated at the angle range of [-15°, 15°] until they met the expected number of training data (1500 slices). However, three hybrid models only used the raw training data (350 slices).

Overall, the hybrid models obtain comparable or better segmentation results than deep learning models. Among all the models, our proposed model has the best performance. All the hybrid models are based on the principal curve and have obtained good segmentation results. These results prove that combining the principal curve model with the machine learning model has the ability to fit the dataset and alleviate the requirement of the large dataset in deep learning-based models

## 5 Discussion and Conclusion

In this paper, we have presented a new hybrid model for accurate and robust prostate segmentation from TRUS images. The innovations of our proposed model include: (1) an improved principal curve model; (2) an improved differential evolution machine learning model; (3) a map mathematical function to generate the smooth prostate contour. To demonstrate the applicability of our proposed segmentation method to prostates of various shapes, TRUS images of 100 patients were used for validating the performance of the proposed method. Both qualitative and quantitative experimental results show that: (1) the H-SegMod can obtain accurate results whether it is validated on different patients (all 25 testing patients) or based on different evaluation metrics (DSC, Ω, and ACC); (2) the performance of the proposed segmentation model outperforms many other state-of-the-art methods. In this section, we discuss the entire study from different aspects.

*The H-SegMod:* This part will be discussed from three aspects: (1) the impact of the selection of optimal parameters of the proposed H-SegMod, (2) discussion of proposed H-SegMod using a different number of seed points, and (3) the worst result of the proposed method.

*The impact of the selection of optimal parameters of the proposed H-SegMod*: In Section 4.1, we present the process of selecting the optimal parameters of the proposed model. From **Figures 3**, **4**, we can find that after the hidden neuron is more than 10 and the epoch is larger than 1000, the performance of ABPNN starts to degrade based on different metrics (i.e., DSC, Ω, and ACC). These could be caused by overfitting, where the excessively increased number of neurons or epochs outweighs the complexity of the ABPNN. Some common strategies, such as standard regularization methods, early stopping, dropout, or data augmentation, may be employed to avoid overfitting (41).

*Discussion of proposed H-SegMod using different number of seed points*: To discuss the impact of number and placement of seed points, we designed two experiments, including seed points closer to the shadowed regions and prostate contour. We used less than 10% of points of manually delineated contour by radiologists as seed points in this work. **Table 5** shows the quantitative comparison of point-guided methods using DSC as a metric, after selecting less than 1%, 4%, 7%, 10%, 15%, 30%, or 50% of points of contours manually delineated by radiologists as the prior points. From **Table 5**, we can find that the DSCs of both methods increase using the increasing number of seed points. Compared with the method using the seed points closer to the shadowed regions, the performance of the method using the seed points closer to the prostate contour increases slightly.

**Table 5:** DSC values of seed point-guided methods using different percentage points as a prior.

Placement of seed points	<1% of points	<4% of points	<7% of points	<10% of points	<15% of points	<30% of points	<50% of points
Closer to the shadowed regions	91	93.1	94.7	**95.7**	96.1	96.6	97.3
Closer to the prostate contour	91.8	93.5	95.8	**96.5**	96.8	97.2	98.5

**Figure 8:**
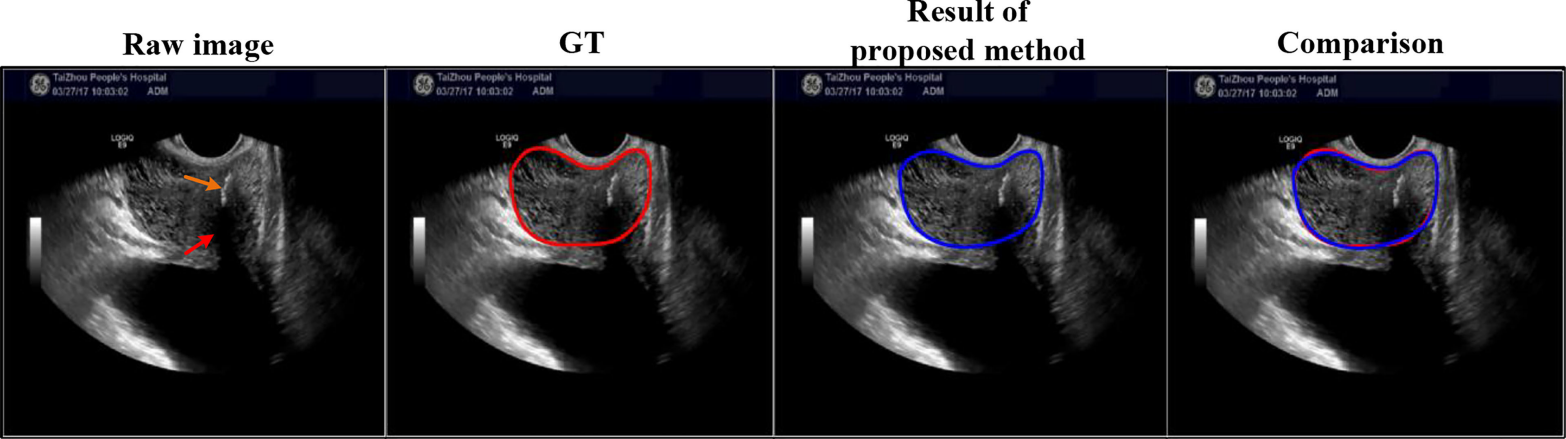
Here is the worst result of the proposed method. Red and orange arrows point to the intraprostatic calcifications and openings of the prostatic urethra, respectively.

*The worst result of the proposed method*: We selected the worst result of the proposed method, shown in **Figure 8**, where the DSC of the proposed method using this case is only 0.79. The prostate is severely affected by intraprostatic calcifications and openings of the prostatic urethra.

*The H-SegMod and curve fitting method*: In Section 4.4, although the curve obtained by CSIM can be close to the approximate trend of the initial data points, the sequence of the initial points needs to be set manually, as shown in **Table 3** and **Figure 7**. In the proposed H-SegMod, the sequence number of the vertices is achieved automatically. Considering that the principal curve f(t) has the ability to pass the center of the data points, the principal curve model can automatically approach the center of the dataset and obtain more accurate results.

*The H-SegMod and state-of-the-art method*: In this section, we discuss four aspects: (1) overall comparison, (2) comparison of the extreme case, (3) computational efficiency, and (4) the degree of difficulty with ultrasound prostate tasks.

*Overall comparison*: Comparison methods described in both the Ref. (38) and Ref. (39) are deep learning models, which obtained reasonable segmentation results. Compared to the proposed hybrid model, the accuracy of segmentation is worse in these two deep learning-based models. One reason is the number of training images is limited in this work. Another reason is that the shapes of the prostate in different patients have high variations, which makes the training of deep learning models difficult.

**Figure 9:**
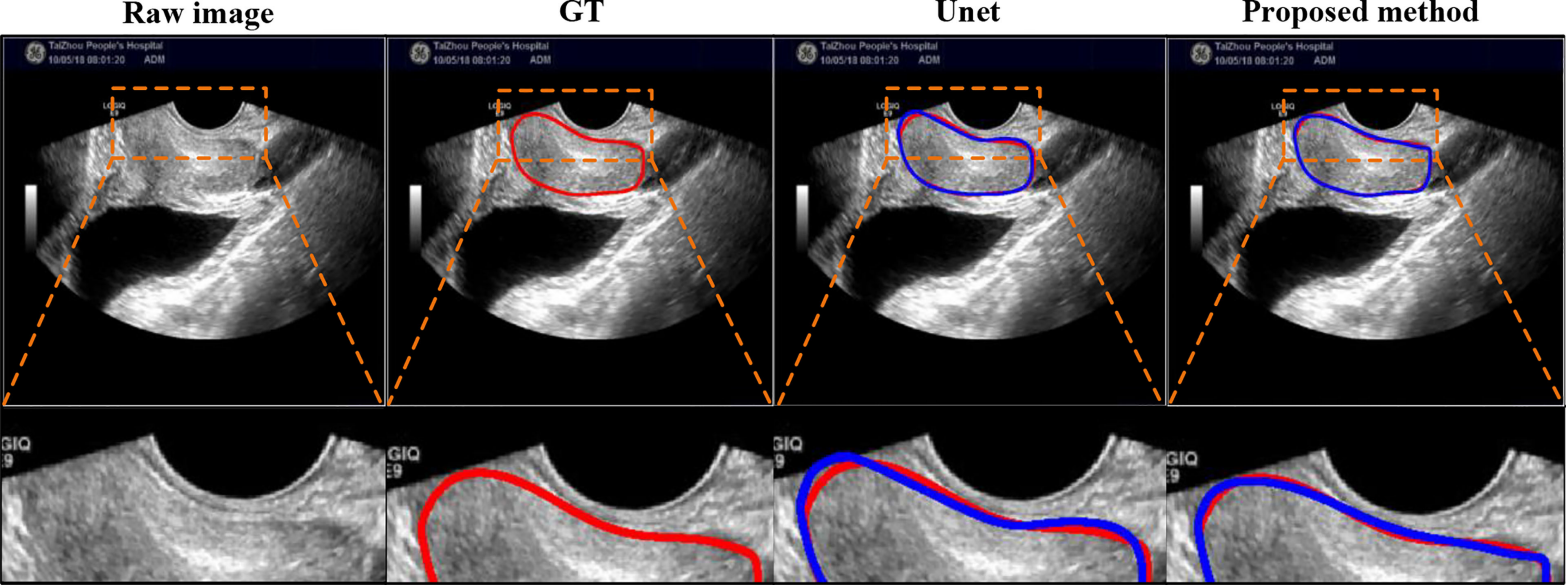
Visual comparison with Unet using extremely case, where the image is mentioned in **Figure 1** (right). The first row shows the compared results, and the second row shows the partial magnification display.

*Comparison on extremely case*: We also added the comparison between Unet and our proposed method on extremely case, shown in **Figure 9**, where this case is mentioned in **Figure 1** (right). This case with the gain being too high causes the weak boundaries between the prostate and neighboring tissues (i.e., bladder and seminal vesicles). From **Figure 9**, our method can obtain a more accurate prostate contour.

*Computational efficiency*: As we know, the performance of deep learning-based methods depends on the amount of training data (42), where we used rotation as the data augmentation stage of the deep learning method. However, this data augmentation strategy is known to cause significant variance in end performance and can be challenging to select (43). The testing times of all the methods in **Table 5** were approximately 6-7s, while there is a big difference in the execution time of all the methods. It spent around 1.5 days for Mask-RCNN’s training, whereas the U-Net method had approximately 3.5 hours for training. The other hybrid methods (CPL-BNNM, Hull-CPLM, and H-ProSeg) spent around 2 hours on training.

*Degree of difficulty with ultrasound prostate tasks*: In **Table 4**, comparison methods described in both Ref. (24) and Ref. (25) are hybrid models. These models were tested on Chest X-rays (CXR) and chest Computed Tomography (CT) for lung segmentation, respectively, and obtained a good performance. However, compared with CXR and CT lung images, TRUS prostate image is more complex and easily affected by other organs such as the urethra, bladder, and rectum. Although lung CXR and CT lung segmentation will be affected by the heart, clavicle, etc., the lung contrast in CXR and CT is higher, which are relatively easier to be identified. In rectal ultrasound prostate segmentation, the boundary of the prostate can be vague. Therefore, the results of hybrid models obtained in this work for prostate TRUS segmentation measured by DSC and ACC slightly degraded as compared to results for lung segmentation on CXR or CT.

While our proposed semi-automatic model has obtained promising results, several aspects can be considered to further improve its performance and develop it into a fully-automatic model. The proposed network architecture consists of two cascaded stages, which increases the memory burden for segmentation. Therefore, model compression needs to be considered for its real-time clinical applications. Furthermore, to develop a fully automatic segmentation method, we may use deep learning models to obtain a coarse segmentation and use the proposed method to refine the coarse segmentation from deep learning.

## 6 Appendix

### 6.1 Notation

**Table 6** shows the description of the used notation.

**Table 6:** A description of the notation used in Appendix A.

	Description	Symbols
**CCPS**	Principal curve	f
	Data point set/data point	P_/_p
	Number of data points	n
	Vertex subset/segment subset in principal curve	V_i_ = {v_1_, v_2_, …, v_iv_}/S_i_ = {s_1_, s_2_, …, s_is_}
	Vertex/segment of principal curve	v/s
	x-axis/y-axis coordinates of principal curve’s vertex v	x/y
	Vertices sequence	t
	Length of segment	l
	Distance deviation	Δs
	Data radius	r
	Flag of vertex cleaning	Flag(•)
**IAMCDE**	Number of populations	S
	Population member	pm
	Individual/new generated individual	Pop/npop
	Trial vector	u
	Present/maximum iteration number	G/Gmax
	Mutation factor/Crossover rate	F/CR
	Predefined lower/upper bounds of the search space	U_min_/U_max_
	Probability of using the mutation operator	pro
**ABPNN**	Number of input neurons	inumber
	Number of hidden neurons	q
	Number of output neurons	k
	Weight from the input layer to the hidden layer	w
	Weight from the hidden layer to the output layer	a
	Threshold of the i-th hidden neuron	T
	Threshold of the u-th output neuron	b
	Output of output units	g(•)

### 6.2 Derivation Procedure of Mathematical Equations

**1. Derivation Procedure of Eq. (15)**

During ABPNN’s training, the output of the output layer is achieved at the forward propagation stage, and the purpose of the backpropagation stage is to update the parameters (i.e., weight and threshold). Sigmoid activation function h_1 =_ 1/(1+e^-x^) and Tanh activation function h_2_=(e^x^-e^-x^)/(e^x^+e^-x^) are used at the forward propagation stage from the input to hidden layer and hidden to output layer, respectively. The details of BPNN are shown in Ref (44). We list the main derivation step of Eq. (15) for making the ABPNN’s procedure intuitive. Furthermore, all the mentioned notations have been summarized in previous Section 6.1.

The input of the hidden layer H_I_ is obtained in Eq. (20).

(20) $${\text{H}}_{\text{I}}=\sum_{\text{i}=1}^{\text{Z}}{\text{tω}}_{\text{i}}-T; i=1,2,\text{..,Z}$$

Sigmoid activation function h_1_ is used to calculate the output of the hidden layer H_O_ (Eq. (21)).

(21) $${\text{H}}_{\text{O}}=\frac{1}{{1+e}^{{\text{-H}}_{\text{I}}}}=\frac{1}{{1+e}^{\sum_{i=1}^{\text{Z}}{\text{-(tω}}_{\text{i}}{\text{-T}}_{\text{i}})}}; i=1,2,\text{..,Z}$$

The input of the output layer g_I_ is shown in Eq. (22), where we set k=2.

(22) $${\text{g}}_{\text{I}}=\sum_{j=1}^{\text{k}}{\text{H}}_{\text{o}}{\text{a}}_{\text{j}}{\text{-b}}_{\text{j}}=\sum_{j=1}^{\text{k}}\frac{1}{{1+e}^{\sum_{i=1}^{\text{q}}{\text{-(tω}}_{\text{i}}{\text{-T}}_{\text{i}})}}{\text{a}}_{\text{j}}{\text{-b}}_{\text{j}}; i=1,2,\text{...},q; j=1,\text{..,k }\left(k=2\right)$$

Furthermore, we used Tanh activation function h_2_ to calculate the output of the output layer g_O_, which is shown in Eq. (23).

(_23)_
$${\text{g}}_{\text{O}}=\frac{{\text{e}}^{{\text{g}}_{\text{I}}}{\text{-e}}^{{\text{-g}}_{\text{I}}}}{{\text{e}}^{{\text{g}}_{\text{I}}}{\text{+e}}^{{\text{-g}}_{\text{I}}}}=\frac{{\text{e}}^{\sum_{j=1}^{\text{k}}\frac{1}{{1+e}^{\sum_{i=1}^{\text{q}}{\text{-(tω}}_{\text{i}}{\text{-T}}_{\text{i}})}}{\text{a}}_{\text{j}}{\text{-b}}_{\text{j}}}{\text{-e}}^{-\sum_{j=1}^{\text{k}}\frac{1}{{1+e}^{\sum_{i=1}^{\text{q}}{\text{-(tω}}_{\text{i}}{\text{-T}}_{\text{i}})}}{\text{a}}_{\text{j}}{\text{-b}}_{\text{j}}}}{{\text{e}}^{\sum_{j=1}^{\text{k}}\frac{1}{{1+e}^{\sum_{i=1}^{\text{q}}{\text{-(tω}}_{\text{i}}{\text{-T}}_{\text{i}})}}{\text{a}}_{\text{j}}{\text{-b}}_{\text{j}}}{\text{+e}}^{-\sum_{j=1}^{\text{k}}\frac{1}{{1+e}^{\sum_{i=1}^{\text{q}}{\text{-(tω}}_{\text{i}}{\text{-T}}_{\text{i}})}}{\text{a}}_{\text{j}}{\text{-b}}_{\text{j}}}}; i=1,2,\ldots ,q; j=1,\text{..,k }\left(k=2\right)$$

where we use the g_o_ to denote the set of two outputs of output layer of ABPNN, and g_o_=[g_x_(t), g_y_(t)]. Furthermore, due to the prostate contour consisting of contour points *v*(*x*, *y*), we use *g_x_* (*t*) and *g_y_* (*t*)} to denote contour points’ x-axis coordinates *x*(*t*) and y-axis coordinates *y*(*t*), respectively.

Therefore, two output neurons g(•) are denoted in Eq. (24), To make the reader more intuitive, we show Eq. (24), where Eq. (15) and Eq. (24) are the same.


(24) $$\left({\text{g}}_{\text{x}}(\text{t}),{\text{g}}_{\text{y}}(\text{t})\right)=(\begin{array}{c} \frac{{\text{e}}^{\sum_{j=1}^{\text{k}}\frac{1}{{1+e}^{\sum_{i=1}^{\text{q}}{\text{-(tω}}_{\text{i}}{\text{-T}}_{\text{i}})}}{\text{a}}_{j,1}{\text{-b}}_{j,1}}{\text{-e}}^{-\sum_{j=1}^{\text{k}}\frac{1}{{1+e}^{\sum_{i=1}^{\text{q}}{\text{-(tω}}_{\text{i}}{\text{-T}}_{\text{i}})}}{\text{a}}_{j,1}{\text{-b}}_{j,1}}}{{\text{e}}^{\sum_{j=1}^{\text{k}}\frac{1}{{1+e}^{\sum_{i=1}^{\text{q}}{\text{-(tω}}_{\text{i}}{\text{-T}}_{\text{i}})}}{\text{a}}_{j,1}{\text{-b}}_{j,1}}{\text{+e}}^{-\sum_{j=1}^{\text{k}}\frac{1}{{1+e}^{\sum_{i=1}^{\text{q}}{\text{-(tω}}_{\text{i}}{\text{-T}}_{\text{i}})}}{\text{a}}_{j,1}{\text{-b}}_{j,1}}},\\ \frac{{\text{e}}^{\sum_{j=1}^{\text{k}}\frac{1}{{1+e}^{\sum_{i=1}^{\text{q}}{\text{-(tω}}_{\text{i}}{\text{-T}}_{\text{i}})}}{\text{a}}_{j,2}{\text{-b}}_{j,2}}{\text{-e}}^{-\sum_{j=1}^{\text{k}}\frac{1}{{1+e}^{\sum_{i=1}^{\text{q}}{\text{-(tω}}_{\text{i}}{\text{-T}}_{\text{i}})}}{\text{a}}_{j,2}{\text{-b}}_{j,2}}}{{\text{e}}^{\sum_{j=1}^{\text{k}}\frac{1}{{1+e}^{\sum_{i=1}^{\text{q}}{\text{-(tω}}_{\text{i}}{\text{-T}}_{\text{i}})}}{\text{a}}_{j,2}{\text{-b}}_{j,2}}{\text{+e}}^{-\sum_{j=1}^{\text{k}}\frac{1}{{1+e}^{\sum_{i=1}^{\text{q}}{\text{-(tω}}_{\text{i}}{\text{-T}}_{\text{i}})}}{\text{a}}_{j,2}{\text{-b}}_{j,2}}} \end{array})$$

**2. Derivation Procedure of Eq. (16)**

In the data mining field, Wang et al. (45) proposed a mathematical function to represent the principal curve of the data set, where activation functions from both the input to the hidden layer and hidden to the output layer are Sigmoid function that is denoted in Eq. (25).


(25) $$\left(\text{x}(\text{t}),\text{y}(\text{t})\right)=(\frac{1}{1+{\text{e}}^{{\text{g}}_{\text{x}}^{'}(\text{t})}},\frac{1}{1+{\text{e}}^{{\text{g}}_{\text{y}}^{'}(\text{t})}})$$

Due to our method using the Sigmoid function from input to hidden layer as well, g’_x_(t) and g’_y_(t) are used as the input of g_x_(t) and g_y_(t), respectively.

Because of its higher training efficiency (46), Tanh activation function from hidden to output layer is used in this study. Based on Tanh activation function, g_x_(t) and g_y_(t) are denoted in Eq. (26) and Eq. (27), respectively.

(26) $${\text{g}}_{\text{x}}\text{(t)=}\frac{{\text{e}}^{{\text{g}}_{x}^{'}\text{(t)}}{\text{-e}}^{{\text{-g}}_{x}^{'}\text{(t)}}}{{\text{e}}^{{\text{g}}_{x}^{'}\text{(t)}}{\text{+e}}^{{\text{-g}}_{x}^{'}\text{(t)}}}=1-\frac{2}{{\text{e}}^{{2g}_{x}^{'}\text{(t)}}+1}\Rightarrow {\text{e}}^{{\text{g}}_{x}^{'}\text{(t)}}=\sqrt{\frac{{1-g}_{\text{x}}\text{(t)}}{{1+g}_{\text{x}}\text{(t)}}}$$

(27) $${\text{g}}_{\text{y}}\text{(t)=}\frac{{\text{e}}^{{\text{g}}_{y}^{'}\text{(t)}}{\text{-e}}^{{\text{-g}}_{y}^{'}\text{(t)}}}{{\text{e}}^{{\text{g}}_{y}^{'}\text{(t)}}{\text{+e}}^{{\text{-g}}_{y}^{'}\text{(t)}}}=1-\frac{2}{{\text{e}}^{{2g}_{y}^{'}\text{(t)}}+1}\Rightarrow {\text{e}}^{{\text{g}}_{y}^{'}\text{(t)}}=\sqrt{\frac{{1-g}_{\text{y}}\text{(t)}}{{1+g}_{\text{y}}\text{(t)}}}$$

After the aforementioned equations [i.e., Eq. (26)~(27)], we use newly obtained ${\text{e}}^{{\text{g}}_{'}^{\text{x}}\text{(t)}}$ and ${\text{e}}^{{\text{g}}_{'}^{\text{y}}\text{(t)}}$ to update the x(t) and y(t) [Eq. (25)]. Finally, we can get the proposed mathematical function of the prostate contour, shown in Eq. (28), where Eq. (16) and Eq. (28) are the same.


(28) $$\text{f}(\text{t})=\left(\text{x}(\text{t)},\text{y}(\text{t})\right)=\left(\frac{{\text{g}}_{\text{x}}(\text{t})+1-\sqrt{1-({\text{g}}_{\text{x}}{\text{(t})}^{2}}}{2*{\text{g}}_{\text{x}}\text{(t)}},\frac{{\text{g}}_{\text{y}}\text{(t)}+1-\sqrt{1-{\left({\text{g}}_{\text{y}}\text{(t})\right)}^{2}}}{2*{\text{g}}_{\text{y}}\text{(t)}}\right)$$

## Data Availability Statement

The data are not publicly available due to the restrictions that contains information that could compromise the privacy of research participants. Requests to access the datasets should be directed to sdpengtao401@gmail.com.

## Ethics Statement

The studies involving human participants were reviewed and approved by Taizhou People’s Hospital. Written informed consent for participation was not required for this study in accordance with the national legislation and the institutional requirements. Written informed consent was not obtained from the individual(s) for the publication of any potentially identifiable images or data included in this article.

## Author Contributions

TP: Methodology, Conceptualization, Formal Analysis, Investigation, Writing-original draft. CT: Investigation, Data curation, Validation, Writing-review &editing. YW: Resources, Investigation, Validation, Writing-review &editing. JC: Supervision, Investigation, Methodology, Writing-review & editing. All authors contributed to the article and approved the submitted version.

## Funding

This work was partly supported by Innovation and Technology Fund Projects, Hong Kong, No. ITS/080/19.

## Conflict of Interest

The authors declare that the research was conducted in the absence of any commercial or financial relationships that could be construed as a potential conflict of interest.

## Publisher’s Note

All claims expressed in this article are solely those of the authors and do not necessarily represent those of their affiliated organizations, or those of the publisher, the editors and the reviewers. Any product that may be evaluated in this article, or claim that may be made by its manufacturer, is not guaranteed or endorsed by the publisher.
